# (3*R*,4*S*)-3,4-Isopropyl­idenedioxy-3,4-dihydro-2*H*-pyrrole 1-oxide

**DOI:** 10.1107/S1600536811013055

**Published:** 2011-04-13

**Authors:** Mari Fe Flores, Pilar Garcia, Narciso M. Garrido, Francisca Sanz, David Diez

**Affiliations:** a Departamento de Quimica Organica, Universidad de Salamanca, Plaza de los Caidos, 37008-Salamanca, Spain; b Servicio General de Rayos X, Universidad de Salamanca, Plaza de los Caidos, 37008-Salamanca, Spain

## Abstract

The title compound C_7_H_11_NO_3_ was prepared by intra­molecular nucleophilic displacement of 2,3-*O*-iso-propyl­idene-d-erythronolactol. There are two mol­ecules in the asymmetric unit, which are related by a pseudo-inversion centre. The crystal structure determination confirms unequivocally the configuration of the chiral centres as 3*S*,4*R*. In the crystal structure, inter­molecular C—H⋯O inter­actions link the mol­ecules (into infinite zigzag chains along the *a* axis.

## Related literature

Nitro­nes play a useful role in the synthesis of complex mol­ec­ular frameworks, undergoing several synthetically useful reactions such as 1,3-dipolar cyclo­additions (Tufariello, 1984 ▶) and nucleophilic addition (Merino *et al.*, 2000 ▶; Lombardo & Trombini, 2002 ▶). They also allow direct access to nitro­nes by simple reactions, see: Döpp & Döpp (1990 ▶); Hamer & Macaluso (1964 ▶). For the use of the title compound as a starting material in the sythesis of potential therapeutic (anti­biotic, anti­viral, anti­tumoral) agents, see: Hall *et al.* (1997 ▶); Closa & Wightman (1998 ▶); McCaig *et al.* (1998 ▶); Cicchi *et al.* (2002 ▶); Revuelta *et al.* (2007 ▶). For a related structure, see: Keleşoğlu *et al.* (2010 ▶). For the preparation of the title compound, see: Flores *et al.* (2010 ▶); Cicchi *et al.* (2006 ▶). 
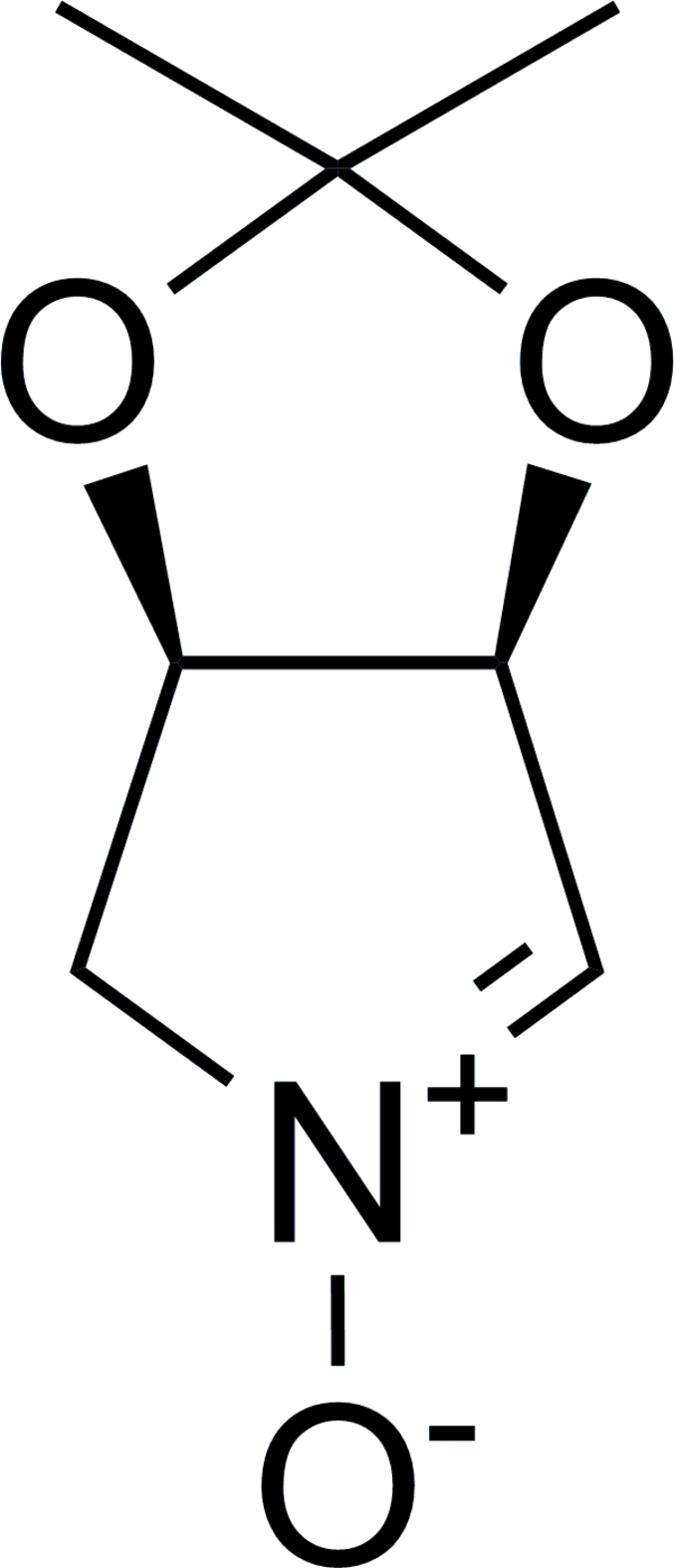


## Experimental

### 

#### Crystal data

C_7_H_11_NO_3_
*M* *_r_* = 157.17
Monoclinic, 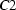

*a* = 11.335 (2) Å
*b* = 5.4467 (11) Å
*c* = 26.508 (5) Å
β = 101.40 (3)°
*V* = 1604.2 (6) Å^3^
*Z* = 8
Cu *K*α radiation
μ = 0.86 mm^−1^
*T* = 298 K
0.15 × 0.10 × 0.08 mm

#### Data collection

Bruker APEXII CCD area-detector diffractometer
Absorption correction: multi-scan (*SADABS*; Bruker, 2006 ▶) *T*_min_ = 0.902, *T*_max_ = 0.934
4369 measured reflections
2365 independent reflections
2261 reflections with *I* > 2σ(*I*)
*R*_int_ = 0.022

#### Refinement

*R*[*F* ^2^ > 2σ(*F* ^2^)] = 0.033
*wR*(*F* ^2^) = 0.091
*S* = 1.05
2365 reflections
204 parameters
1 restraint
H-atom parameters constrained
Δρ_max_ = 0.16 e Å^−3^
Δρ_min_ = −0.11 e Å^−3^
Absolute structure: Flack (1983 ▶), 761 Friedel pairs
Flack parameter: 0.0 (2)

### 

Data collection: *APEX2* (Bruker, 2006 ▶); cell refinement: *SAINT* (Bruker, 2006 ▶); data reduction: *SAINT*; program(s) used to solve structure: *SHELXS97* (Sheldrick, 2008 ▶); program(s) used to refine structure: *SHELXL97* (Sheldrick, 2008 ▶); molecular graphics: *Mercury* (Macrae *et al.*, 2006 ▶); software used to prepare material for publication: *SHELXL97*.

## Supplementary Material

Crystal structure: contains datablocks global, I. DOI: 10.1107/S1600536811013055/bt5507sup1.cif

Structure factors: contains datablocks I. DOI: 10.1107/S1600536811013055/bt5507Isup2.hkl

Additional supplementary materials:  crystallographic information; 3D view; checkCIF report

## Figures and Tables

**Table 1:** Hydrogen-bond geometry (Å, °)

*D*—H⋯*A*	*D*—H	H⋯*A*	*D*⋯*A*	*D*—H⋯*A*
C3—H3⋯O1^i^	0.93	2.44	3.366 (3)	171
C1—H1⋯O3^ii^	0.98	2.38	3.355 (3)	171
C2—H2*A*⋯O3^iii^	0.97	2.70	3.441 (3)	134
C2—H2*B*⋯O3^iv^	0.97	2.41	3.120 (3)	130
C2′—H2′1⋯O2′^v^	0.97	2.49	3.247 (2)	135
C4′—H4′⋯O3′^vi^	0.98	2.48	3.375 (3)	152
C2′—H2′2⋯O3′^vii^	0.97	2.61	3.254 (2)	124
C3′—H3′⋯O3′^viii^	0.93	2.48	3.345 (3)	156

Symmetry codes: (i) 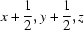; (ii) 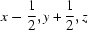; (iii) 
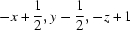
; (iv) 
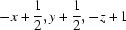
; (v) 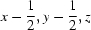; (vi) 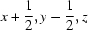; (vii) 
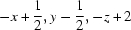
; (viii) 
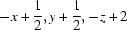
.

**Fig. 1.**
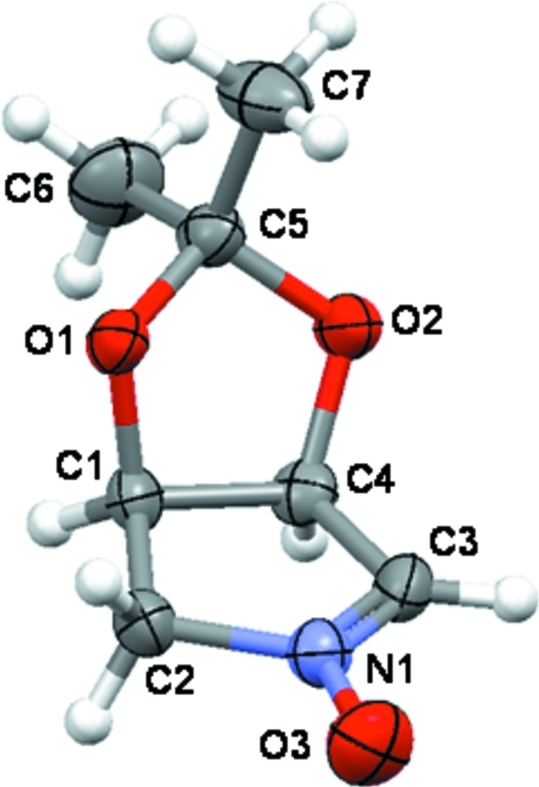
Molecular structure of C7H11NO3.

**Fig. 2.**
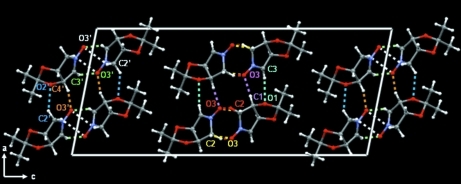
Crystal packing of C7H11NO3 view along b axis, showing intermolecular hydrogen bonding.

## supplementary crystallographic information

### Comment

Nitrones have been the subject of intense research efforts, because of the wide role played in the synthesis of complex molecular frameworks. They undergo several synthetically useful reactions such as 1,3-dipolar cycloadditions, (Tufariello *et al.*, 1984) nucleophilic additions, (Merino *et al.*, 2000; Lombardo *et al.*, 2002). Both the reactions give rise to the formation of new carbon-carbon bonds, often with a high degree of stereocontrol. These features, together with the direct access to nitrones by simple reactions (Hamer *et al.*, 1964; Döpp *et al.*, 1990), and their stability which permits isolation and long storage, make nitrones ideal tools for application in organic syntheses, particularly in the field of alkaloids, nitrogen containing natural products or bioactive analogues. The construction of highly functionalized nitrogen heterocycles in a stereoselective manner is an important focus of medicinal and natural product chemistry. Although, in the last few years, the title compound has been reported more and more in the literature as a starting material due to its biological relevance in the synthesis of polyhydroxypyrrolidines or polyhydroxypyrrolizidines, both interesting compounds as potential glycosidase inhibitors and consequently as potential therapeutic (antibiotic, antiviral, antitumoral) agents (Hall *et al.*, 1997; Closa *et al.*, 1998; McCaig *et al.*, 1998; Cicchi *et al.*, 2002; Revuelta *et al.*, 2007) there was not any crystallographic data.

Following our special interest in nitrogen compounds such as isoxazolidines, we prepared the title N-oxide, and its crystal structure is reported here.

The asymmetric unit contains two symmetrically independent molecules. The title molecule consists of a pyrroline-N-oxide ring with an isopropylidenedioxy as substituent. All the bond lengths and angles are within the normal ranges. The carbonyl group at N1 is coplanar with the pyrroline ring being the O3—N1=C3—C4 and O3'-N1'=C3'-C4' torsion angles of 179.1 (4)° and 179.2 (7)°, respectively. These results are in good agreement with the literature (Keleşoğlu *et al.*, 2010).

In the crystal structure, intermolecular C—H···O interactions (Table 1) link the molecules (Fig. 2) into infinite zigzag chains along the *a* axis.

### Experimental

The title N-oxide  was obtained by intramolecular nucleophilic displacement, which is based on a simple one-pot procedure employing NH_2_OSiMe_2_*t*-Bu, methanesulfonyl chloride, and 2,3-*O*-iso-propylidene-D-erythronolactol, according to the methodology described by Cicchi *et al.* (2006) and by us (Flores *et al.*, 2010). Well shaped colourless single crystals were obtained by crystallization from CH_2_Cl_2_/MeOH.

### Refinement

Hydrogen atoms were positioned geometrically, with C—H distances constrained to 0.93 Å (aromatic CH), 0.96 Å (methyl), 0.97 Å (methylene) and 0.98 Å (methine) and refined in riding mode with *U*_iso_(H) = xUeq(C), where *x* = 1.5 for methyl H atoms and *x* = 1.2 for all other atoms.

### Crystal data

**Table:**

C_7_H_11_NO_3_	*F*(000) = 672
*M**_r_* = 157.17	*D*_x_ = 1.302 Mg m^−^^3^
Monoclinic, *C*2	Cu *K*α radiation, λ = 1.54178 Å
Hall symbol: C 2y	Cell parameters from 2365 reflections
*a* = 11.335 (2) Å	θ = 1.7–66.9°
*b* = 5.4467 (11) Å	µ = 0.86 mm^−^^1^
*c* = 26.508 (5) Å	*T* = 298 K
β = 101.40 (3)°	Prismatic, colourless
*V* = 1604.2 (6) Å^3^	0.15 × 0.10 × 0.08 mm
*Z* = 8	

### Data collection

**Table:**

Bruker APEXII CCD area-detector diffractometer	2365 independent reflections
Radiation source: fine-focus sealed tube	2261 reflections with *I* > 2σ(*I*)
graphite	*R*_int_ = 0.022
phi and ω scans	θ_max_ = 66.9°, θ_min_ = 1.7°
Absorption correction: multi-scan (*SADABS*; Bruker, 2006)	*h* = −13→11
*T*_min_ = 0.902, *T*_max_ = 0.934	*k* = −6→5
4369 measured reflections	*l* = −28→31

### Refinement

**Table:**

Refinement on *F*^2^	Hydrogen site location: inferred from neighbouring sites
Least-squares matrix: full	H-atom parameters constrained
*R*[*F*^2^ > 2σ(*F*^2^)] = 0.033	*w* = 1/[σ^2^(*F*_o_^2^) + (0.0479*P*)^2^ + 0.4187*P*] where *P* = (*F*_o_^2^ + 2*F*_c_^2^)/3
*wR*(*F*^2^) = 0.091	(Δ/σ)_max_ = 0.001
*S* = 1.05	Δρ_max_ = 0.16 e Å^−^^3^
2365 reflections	Δρ_min_ = −0.11 e Å^−^^3^
204 parameters	Extinction correction: *SHELXL*, Fc^*^=kFc[1+0.001xFc^2^λ^3^/sin(2θ)]^-1/4^
1 restraint	Extinction coefficient: 0.00105 (18)
Primary atom site location: structure-invariant direct methods	Absolute structure: Flack (1983), 761 Friedel pairs
Secondary atom site location: difference Fourier map	Flack parameter: 0.0 (2)

### Special details

**Table:**

Geometry. All e.s.d.'s (except the e.s.d. in the dihedral angle between two l.s. planes) are estimated using the full covariance matrix. The cell e.s.d.'s are taken into account individually in the estimation of e.s.d.'s in distances, angles and torsion angles; correlations between e.s.d.'s in cell parameters are only used when they are defined by crystal symmetry. An approximate (isotropic) treatment of cell e.s.d.'s is used for estimating e.s.d.'s involving l.s. planes.
Refinement. Refinement of *F*^2^ against ALL reflections. The weighted *R*-factor *wR* and goodness of fit *S* are based on *F*^2^, conventional *R*-factors *R* are based on *F*, with *F* set to zero for negative *F*^2^. The threshold expression of *F*^2^ > σ(*F*^2^) is used only for calculating *R*-factors(gt) *etc*. and is not relevant to the choice of reflections for refinement. *R*-factors based on *F*^2^ are statistically about twice as large as those based on *F*, and *R*- factors based on ALL data will be even larger.

### Fractional atomic coordinates and isotropic or equivalent isotropic displacement parameters (Å^2^)

**Table:**

	*x*	*y*	*z*	*U*_iso_*/*U*_eq_	
O1'	0.30975 (12)	0.5460 (3)	0.84350 (5)	0.0571 (4)	
O2'	0.44334 (11)	0.8279 (3)	0.88116 (5)	0.0566 (4)	
O3'	0.14788 (12)	0.7976 (4)	0.96810 (6)	0.0693 (5)	
N1'	0.23631 (12)	0.7223 (3)	0.94785 (5)	0.0481 (4)	
C1'	0.33483 (16)	0.4762 (4)	0.89604 (7)	0.0476 (4)	
H1'	0.3729	0.3142	0.9013	0.057*	
C3'	0.34095 (15)	0.8196 (4)	0.95186 (6)	0.0464 (4)	
H3'	0.3657	0.9601	0.9709	0.056*	
C4'	0.41764 (14)	0.6811 (4)	0.92243 (6)	0.0463 (4)	
H4'	0.4907	0.6168	0.9445	0.056*	
C5'	0.40764 (17)	0.6940 (4)	0.83486 (7)	0.0525 (5)	
C6'	0.3627 (3)	0.8714 (6)	0.79227 (9)	0.0866 (8)	
H6'1	0.2985	0.9674	0.8009	0.130*	
H6'2	0.3337	0.7826	0.7610	0.130*	
H6'3	0.4271	0.9780	0.7876	0.130*	
C7'	0.5109 (3)	0.5377 (6)	0.82529 (12)	0.0928 (9)	
H7'1	0.4860	0.4458	0.7941	0.139*	
H7'2	0.5349	0.4266	0.8536	0.139*	
H7'3	0.5776	0.6413	0.8221	0.139*	
C2'	0.22244 (16)	0.4919 (4)	0.91839 (8)	0.0553 (5)	
H2'1	0.1510	0.4965	0.8913	0.066*	
H2'2	0.2168	0.3528	0.9406	0.066*	
O1	0.09613 (11)	0.3663 (3)	0.62016 (5)	0.0557 (4)	
O2	0.21761 (13)	0.6589 (3)	0.66182 (5)	0.0666 (5)	
O3	0.36792 (14)	0.3733 (4)	0.52993 (7)	0.0861 (6)	
N1	0.29356 (13)	0.4731 (4)	0.55436 (6)	0.0544 (4)	
C1	0.10961 (15)	0.5348 (4)	0.58059 (7)	0.0514 (5)	
H1	0.0341	0.6186	0.5659	0.062*	
C2	0.16439 (16)	0.4088 (4)	0.54033 (7)	0.0556 (5)	
H2A	0.1530	0.2325	0.5413	0.067*	
H2B	0.1293	0.4687	0.5062	0.067*	
C3	0.31507 (17)	0.6385 (4)	0.58935 (8)	0.0586 (5)	
H3	0.3910	0.7049	0.6014	0.070*	
C4	0.20620 (17)	0.7127 (4)	0.60855 (7)	0.0522 (5)	
H4	0.1847	0.8848	0.6007	0.063*	
C5	0.12362 (18)	0.4967 (4)	0.66756 (7)	0.0558 (5)	
C6	0.0151 (3)	0.6365 (7)	0.67681 (11)	0.0971 (10)	
H6A	−0.0464	0.5228	0.6816	0.146*	
H6B	−0.0148	0.7394	0.6477	0.146*	
H6C	0.0374	0.7365	0.7070	0.146*	
C7	0.1714 (3)	0.3142 (6)	0.70956 (9)	0.0882 (8)	
H7A	0.2404	0.2320	0.7016	0.132*	
H7B	0.1101	0.1958	0.7120	0.132*	
H7C	0.1942	0.3990	0.7418	0.132*	

### Atomic displacement parameters (Å^2^)

**Table:**

	*U*^11^	*U*^22^	*U*^33^	*U*^12^	*U*^13^	*U*^23^
O1'	0.0642 (8)	0.0626 (10)	0.0440 (7)	−0.0248 (7)	0.0098 (6)	−0.0060 (6)
O2'	0.0573 (7)	0.0637 (9)	0.0517 (7)	−0.0225 (7)	0.0178 (6)	−0.0098 (7)
O3'	0.0556 (7)	0.0876 (12)	0.0713 (9)	0.0236 (8)	0.0283 (7)	0.0071 (9)
N1'	0.0431 (7)	0.0554 (10)	0.0467 (8)	0.0091 (7)	0.0108 (6)	0.0067 (7)
C1'	0.0541 (9)	0.0433 (10)	0.0471 (9)	0.0010 (9)	0.0142 (8)	0.0032 (8)
C3'	0.0484 (9)	0.0476 (10)	0.0420 (9)	−0.0002 (8)	0.0061 (7)	0.0000 (8)
C4'	0.0374 (8)	0.0565 (12)	0.0434 (9)	0.0022 (8)	0.0042 (7)	0.0000 (9)
C5'	0.0612 (11)	0.0527 (12)	0.0467 (10)	−0.0157 (9)	0.0176 (8)	−0.0056 (9)
C6'	0.115 (2)	0.0827 (19)	0.0616 (13)	−0.0206 (17)	0.0163 (13)	0.0152 (14)
C7'	0.1036 (19)	0.084 (2)	0.108 (2)	0.0040 (17)	0.0634 (17)	−0.0118 (18)
C2'	0.0509 (10)	0.0578 (12)	0.0581 (11)	−0.0114 (10)	0.0127 (8)	−0.0032 (10)
O1	0.0564 (7)	0.0588 (9)	0.0530 (7)	−0.0180 (7)	0.0137 (6)	−0.0052 (7)
O2	0.0758 (9)	0.0742 (12)	0.0499 (8)	−0.0306 (9)	0.0126 (6)	−0.0074 (8)
O3	0.0679 (9)	0.1030 (15)	0.0940 (11)	0.0252 (10)	0.0316 (8)	−0.0034 (12)
N1	0.0472 (8)	0.0598 (10)	0.0560 (9)	0.0068 (8)	0.0098 (7)	0.0045 (9)
C1	0.0395 (8)	0.0617 (14)	0.0502 (10)	0.0022 (9)	0.0022 (7)	0.0044 (9)
C2	0.0511 (10)	0.0646 (14)	0.0488 (10)	−0.0048 (9)	0.0045 (8)	−0.0025 (10)
C3	0.0465 (9)	0.0662 (14)	0.0612 (11)	−0.0129 (9)	0.0061 (8)	0.0058 (11)
C4	0.0600 (11)	0.0397 (10)	0.0572 (11)	−0.0048 (9)	0.0127 (9)	0.0008 (9)
C5	0.0627 (11)	0.0549 (12)	0.0523 (10)	−0.0128 (10)	0.0179 (8)	−0.0059 (10)
C6	0.0920 (19)	0.112 (3)	0.098 (2)	0.0096 (19)	0.0441 (16)	−0.018 (2)
C7	0.127 (2)	0.0758 (18)	0.0603 (13)	−0.0100 (18)	0.0159 (13)	0.0090 (14)

### Geometric parameters (Å, °)

**Table:**

O1'—C1'	1.417 (2)	O1—C5	1.423 (2)
O1'—C5'	1.426 (2)	O1—C1	1.425 (2)
O2'—C5'	1.416 (2)	O2—C5	1.415 (2)
O2'—C4'	1.431 (2)	O2—C4	1.423 (2)
O3'—N1'	1.2937 (19)	O3—N1	1.281 (2)
N1'—C3'	1.284 (2)	N1—C3	1.282 (3)
N1'—C2'	1.470 (3)	N1—C2	1.480 (2)
C1'—C2'	1.510 (2)	C1—C2	1.502 (3)
C1'—C4'	1.535 (3)	C1—C4	1.538 (3)
C1'—H1'	0.9800	C1—H1	0.9800
C3'—C4'	1.484 (3)	C2—H2A	0.9700
C3'—H3'	0.9300	C2—H2B	0.9700
C4'—H4'	0.9800	C3—C4	1.481 (3)
C5'—C6'	1.497 (3)	C3—H3	0.9300
C5'—C7'	1.509 (4)	C4—H4	0.9800
C6'—H6'1	0.9600	C5—C6	1.507 (3)
C6'—H6'2	0.9600	C5—C7	1.511 (4)
C6'—H6'3	0.9600	C6—H6A	0.9600
C7'—H7'1	0.9600	C6—H6B	0.9600
C7'—H7'2	0.9600	C6—H6C	0.9600
C7'—H7'3	0.9600	C7—H7A	0.9600
C2'—H2'1	0.9700	C7—H7B	0.9600
C2'—H2'2	0.9700	C7—H7C	0.9600
			
C1'—O1'—C5'	107.35 (14)	C5—O1—C1	106.97 (16)
C5'—O2'—C4'	107.96 (15)	C5—O2—C4	108.22 (15)
C3'—N1'—O3'	127.85 (19)	O3—N1—C3	127.89 (18)
C3'—N1'—C2'	113.34 (15)	O3—N1—C2	119.33 (19)
O3'—N1'—C2'	118.74 (16)	C3—N1—C2	112.67 (16)
O1'—C1'—C2'	110.46 (15)	O1—C1—C2	110.37 (18)
O1'—C1'—C4'	103.80 (15)	O1—C1—C4	102.75 (14)
C2'—C1'—C4'	105.50 (15)	C2—C1—C4	105.98 (15)
O1'—C1'—H1'	112.2	O1—C1—H1	112.4
C2'—C1'—H1'	112.2	C2—C1—H1	112.4
C4'—C1'—H1'	112.2	C4—C1—H1	112.4
N1'—C3'—C4'	111.98 (18)	N1—C2—C1	103.94 (16)
N1'—C3'—H3'	124.0	N1—C2—H2A	111.0
C4'—C3'—H3'	124.0	C1—C2—H2A	111.0
O2'—C4'—C3'	110.31 (17)	N1—C2—H2B	111.0
O2'—C4'—C1'	104.86 (13)	C1—C2—H2B	111.0
C3'—C4'—C1'	103.90 (14)	H2A—C2—H2B	109.0
O2'—C4'—H4'	112.4	N1—C3—C4	112.86 (17)
C3'—C4'—H4'	112.4	N1—C3—H3	123.6
C1'—C4'—H4'	112.4	C4—C3—H3	123.6
O2'—C5'—O1'	104.48 (13)	O2—C4—C3	111.49 (17)
O2'—C5'—C6'	108.5 (2)	O2—C4—C1	105.37 (15)
O1'—C5'—C6'	109.05 (19)	C3—C4—C1	102.97 (17)
O2'—C5'—C7'	109.8 (2)	O2—C4—H4	112.2
O1'—C5'—C7'	111.2 (2)	C3—C4—H4	112.2
C6'—C5'—C7'	113.4 (2)	C1—C4—H4	112.2
C5'—C6'—H6'1	109.5	O2—C5—O1	104.75 (13)
C5'—C6'—H6'2	109.5	O2—C5—C6	111.0 (2)
H6'1—C6'—H6'2	109.5	O1—C5—C6	110.64 (19)
C5'—C6'—H6'3	109.5	O2—C5—C7	108.8 (2)
H6'1—C6'—H6'3	109.5	O1—C5—C7	107.8 (2)
H6'2—C6'—H6'3	109.5	C6—C5—C7	113.4 (2)
C5'—C7'—H7'1	109.5	C5—C6—H6A	109.5
C5'—C7'—H7'2	109.5	C5—C6—H6B	109.5
H7'1—C7'—H7'2	109.5	H6A—C6—H6B	109.5
C5'—C7'—H7'3	109.5	C5—C6—H6C	109.5
H7'1—C7'—H7'3	109.5	H6A—C6—H6C	109.5
H7'2—C7'—H7'3	109.5	H6B—C6—H6C	109.5
N1'—C2'—C1'	104.30 (16)	C5—C7—H7A	109.5
N1'—C2'—H2'1	110.9	C5—C7—H7B	109.5
C1'—C2'—H2'1	110.9	H7A—C7—H7B	109.5
N1'—C2'—H2'2	110.9	C5—C7—H7C	109.5
C1'—C2'—H2'2	110.9	H7A—C7—H7C	109.5
H2'1—C2'—H2'2	108.9	H7B—C7—H7C	109.5

### Hydrogen-bond geometry (Å, °)

**Table:**

*D*—H···*A*	*D*—H	H···*A*	*D*···*A*	*D*—H···*A*
C3—H3···O1^i^	0.93	2.44	3.366 (3)	171
C1—H1···O3^ii^	0.98	2.38	3.355 (3)	171
C2—H2A···O3^iii^	0.97	2.70	3.441 (3)	134
C2—H2B···O3^iv^	0.97	2.41	3.120 (3)	130
C2'—H2'1···O2'^v^	0.97	2.49	3.247 (2)	135
C4'—H4'···O3'^vi^	0.98	2.48	3.375 (3)	152
C2'—H2'2···O3'^vii^	0.97	2.61	3.254 (2)	124
C3'—H3'···O3'^viii^	0.93	2.48	3.345 (3)	156

Symmetry codes: (i) *x*+1/2, *y*+1/2, *z*; (ii) *x*−1/2, *y*+1/2, *z*; (iii) −*x*+1/2, *y*−1/2, −*z*+1; (iv) −*x*+1/2, *y*+1/2, −*z*+1; (v) *x*−1/2, *y*−1/2, *z*; (vi) *x*+1/2, *y*−1/2, *z*; (vii) −*x*+1/2, *y*−1/2, −*z*+2; (viii) −*x*+1/2, *y*+1/2, −*z*+2.
